# Influence of organisational culture on the implementation of health sector reforms in low- and middle-income countries: a qualitative interpretive review

**DOI:** 10.1080/16549716.2018.1462579

**Published:** 2018-05-11

**Authors:** Rahab Mbau, Lucy Gilson

**Affiliations:** a School of Public Health and Family Medicine, University of Cape Town, Cape Town, South Africa; b Health Policy and Systems Division, School of Public Health and Family Medicine, University of Cape Town, Cape Town, South Africa; c Department of Global Health and Development, Faculty of Public Health and Policy, London School of Hygiene and Tropical Medicine, London, UK

**Keywords:** Organisational culture, health sector reforms, implementation, low- and middle-income countries, qualitative interpretive synthesis

## Abstract

**Background**: Health systems, particularly in low- and middle-income countries, are commonly plagued by poor access, poor performance, inefficient use and inequitable distribution of resources. To improve health system efficiency, equity and effectiveness, the World Development Report of 1993 proposed a first wave of health sector reforms, which has been followed by further waves. Various authors, however, suggest that the early reforms did not lead to the anticipated improvements. They offer, as one plausible explanation for this gap, the limited consideration given to the influence over implementation of the software aspects of the health system, such as organisational culture – which has not previously been fully investigated.

**Objective**: To identify, interpret and synthesise existing literature for evidence on organisational culture and how it influences implementation of health sector reforms in low- and middle-income countries.

**Methods**: We conducted a systematic search of eight databases: PubMed; Africa-Wide Information, Cumulative Index of Nursing and Allied Health Literature (CINAHL), Econlit, PsycINFO, SocINDEX with full text, Emerald and Scopus. Eight papers were identified. We analysed and synthesised these papers using thematic synthesis.

**Results**: This review indicates the potential influence of dimensions of organisational culture such as power distance, uncertainty avoidance, and in-group and institutional collectivism over the implementation of health sector reforms. This influence is mediated through organisational practices such as communication and feedback, management styles, commitment and participation in decision-making.

**Conclusion**: This interpretive review highlights the dearth of empirical literature around organisational culture and therefore its findings can only be tentative. There is a need for health policymakers and health system researchers to conduct further analysis of organisational culture and change within the health system.

## Background

Health systems, particularly in low- and middle-Income countries (LMICs), are commonly plagued by problems such as poor access and performance, as well as inefficient use, and inequitable distribution, of resources [1–3]. Recognising that such problems reflect system-wide deficiencies rather than weaknesses in particular health programmes or areas of service delivery, the World Bank proposed a first wave of ‘health sector reforms’, with the goals of improving health system efficiency, equity and effectiveness, in the World Development Report of 1993 [4]. The reforms emphasised at this time were decentralisation, user fees and social health insurance, pay for performance, public–private partnerships, contracting out of health services, and comprehensive primary health care [1,4–6]. Subsequent waves of similar reforms have been called for under the banner ‘health system strengthening’ [7] and, most recently, Universal Health Coverage [8]. For the most part, the reforms of focus largely address the hardware elements of the health system [9] – that is, the tangible and functional aspects of the system [10] that make up its basic building blocks: service delivery, health care financing, health workforce, leadership and governance, information, and medical products, vaccines and technology [11].

The changes resulting from the mid-1990s reforms were, however, varied [3], with authors such as Blaauw et al. [9] suggesting that the gains achieved were limited. Some authors specifically suggested that challenges resulted from the influence of health system software on reform implementation [9,12,13]. The software elements of the health system refer to the intangible aspects that govern functions and relationships within the health system such as ideas, values, interests, power and norms [10] as well as organisational culture [9].

Indeed, since the early 2000s, organisational culture has been a key theme of debate in relation to the structural, or hardware, reforms in the health sector within high-income countries such as the USA and the UK. Policymakers and managers in these countries realised that structural reforms on their own cannot lead to the desired changes within the health systems [12–16]. Organisational culture is specifically noted as having the potential to shape the way that such reforms are put into action [12]. Whilst there are numerous definitions of organisational culture, all are based on a view of organisations as social systems characterised by social processes, behaviours and structures [17,18] and all understand culture to be shared social constructs such as beliefs, meanings, values, behaviour and norms [15,19,20]. Within a single organisation there may be several cultures linked to particular subgroups or units, and organisational culture is itself influenced by broader societal factors, including history and political culture [21].

In view of the potential importance of organisational culture as an influence on the implementation of health sector reforms at local level in LMICs, this qualitative synthesis was undertaken to take stock of the current knowledge base, to draw on relevant research, and, if possible, policy implications. In this aim it was in line with other, recent qualitative synthesis work (special issue of *Health Policy and Planning* 29[3], December 2014). It reviews existing empirical literature from LMICs to identify, interpret and synthesise evidence on organisational culture and its influence on the implementation of such reforms. The core review question is ‘How does organisational culture influence the implementation of health sector reforms in LMICs?’ In this synthesis, organisational culture is conceptualised as a system of values and practices that are socially constructed and shared by actors and influence their relationships, attitudes and behaviour towards changes, and can be manipulated or influenced, at least in part, through managerial strategies to enable achievement of the desired organisational goals. Practices refer to how things are done while values refer to judgements of how things should be done [22]. An organisation refers to a structured and formalised entity made up of a group of people who have come together for a common purpose [23].

## Methods

This review employed an interpretive qualitative synthesis approach to interpret and synthesise findings from all forms of empirical studies whether qualitative, quantitative or mixed methods. Although founded on the principles of systematic review [24,25], such an approach goes beyond a review of literature to generate new concepts and meanings from synthesis of the collated work [25,26]. These new ideas are, in essence, analytic generalisations of potential relevance in settings beyond those specifically considered in the papers reviewed [27].

### Literature search

**Figure 1.**
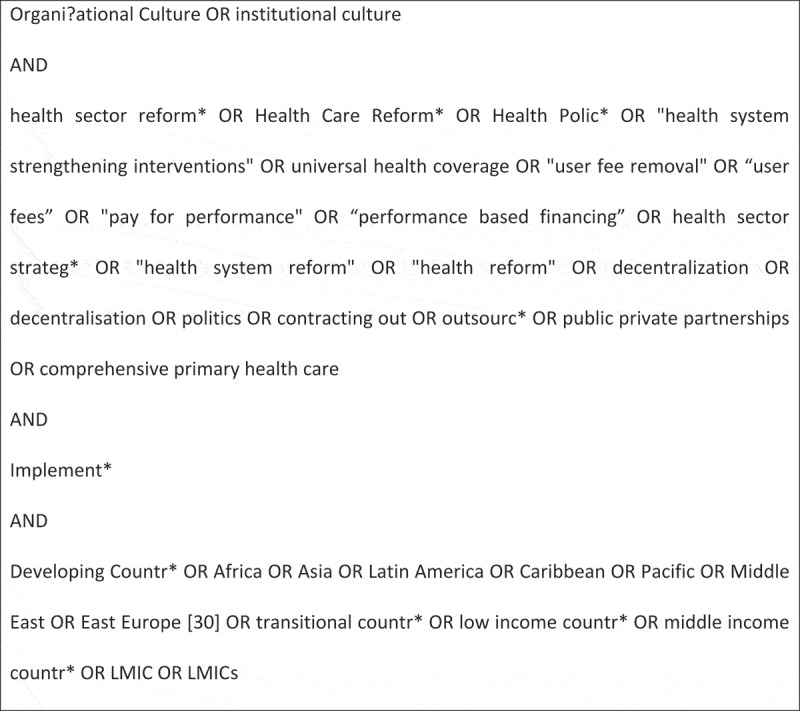
Key search terms.

We conducted a systematic electronic database search using key search terms (Figure 1) derived from the main concepts in the review question and strengthened by broader literature as well as consultations and inputs from LG (co-author), an experienced health policy and health systems researcher. The development of the search string and the subsequent literature search involved an iterative process that was done under the skilful assistance of a health science librarian from the University of Cape Town. We used eight databases that we considered relevant to the review due to their focus and accessibility to the primary reviewer. These databases included: PubMed, Africa-Wide Information, Cumulative Index of Nursing and Allied Health Literature (CINAHL), Econlit, PsycINFO, SocINDEX with full text, Emerald and Scopus. Multiple databases were included to minimise selection bias [28]. The initial search was conducted in PubMed and then translated to the other databases according to their appropriate controlled vocabulary and standardised terms of indexing [28]. To be as comprehensive as possible, the initial search in PubMed was carried out using country-specific names according to the 2012 LMICs filters developed by the Norwegian satellite of the Cochrane Effective Practice and Organisation of Care Group [29]. A comprehensive account of the literature search strategy used for each database and the dates of the last search are provided in the text in Supplementary material 1.

### Inclusion criteria

We identified potentially relevant articles by first removing the articles that were not related to the health sector and those that had a high-income country focus. We downloaded the remaining papers into a data reference manager, RefWorks, for easier data management and removal of duplicates [28]. We then removed the duplicates and reviewed the titles and abstracts of the remaining articles against the inclusion criteria. We only included articles that were published in English, articles with full access, articles with a focus on LMICs, articles whose titles and abstracts contained the keywords and were relevant to the review question, articles published between 1 January 2000 and 31 December 2015, and articles with an empirical focus including qualitative, quantitative and mixed methods. The year 2000 was chosen as the start date for the search because it was the time when organisational culture came to be acknowledged as a potential influence over health sector reform implementation [12].

## Quality appraisal

We included all the articles that met the inclusion criteria in this review irrespective of their quality. This approach recognised the limited number of articles retrieved and that, following the Cochrane Qualitative Research Methods Group [31], the value of each study may only become apparent in the synthesis rather than at the point of appraisal.

### Extraction and synthesis of data

We extracted data from all the sections of the articles given that different reporting styles across academic disciplines means that relevant data may be presented in sections other than the findings section alone [32]. We used the thematic synthesis approach to analyse and synthesise the data [33]. RM initially read each of the articles line by line and identified and coded the texts, quotes and authorial judgments. Following Gilson et al. [34], authorial judgements were included as data because they offered more insight into the data presented in the studies. In an inductive process, we merged similar codes into descriptive themes related to values and practices and, where differences were found, these were resolved by consensus. These themes are presented in the Findings section. We then used the House et al. [22] dimensions of organisational culture (Table 1) to deductively interpret and synthesise the findings of the review. We selected this conceptual framework over others, such as Schein’s model [19], the culture web framework [35], Hofstede’s dimensions of organisational culture [36] and the competing values framework [21], for two reasons: (1) it had been piloted and tested across different sectors (telecommunication, finance and food processing sectors) in both higher- and lower-income countries, suggesting it was suitable for cross-cultural analysis [22]; (2) it both builds on the dimensions of organisational culture described by others scholars and reflects broader societal influences over its dimensions [22].

**Table 1.** House et al. dimensions of organisational culture.

Dimension	Definition
Power distance	Extent of distribution and concentration of power across the organisation or the society
Institutional collectivism	Extent to which the organisation or society encourages and rewards communal action and sharing of resources
In-group collectivism	Level of pride, satisfaction and loyalty shown by members towards their organisation or society
Uncertainty avoidance	Degree to which the members of an organisation or society avoid unknown circumstances or uncertainty by depending on accepted practices, rules or procedures
Gender egalitarianism	Extent to which the organisation or society minimises differences in roles and opportunities based on gender.
Aggressiveness	Extent to which members of an organisation or society are competitive and confrontational with each other
Humane orientation	Extent to which an organisation or society encourages and rewards altruistic behaviour
Future orientation	Extent to which organisations or societies develop plans and strategies for future
Performance orientation	Extent to which the organisation or society encourages excellence and awards improvement

## Results

**Figure 2.**
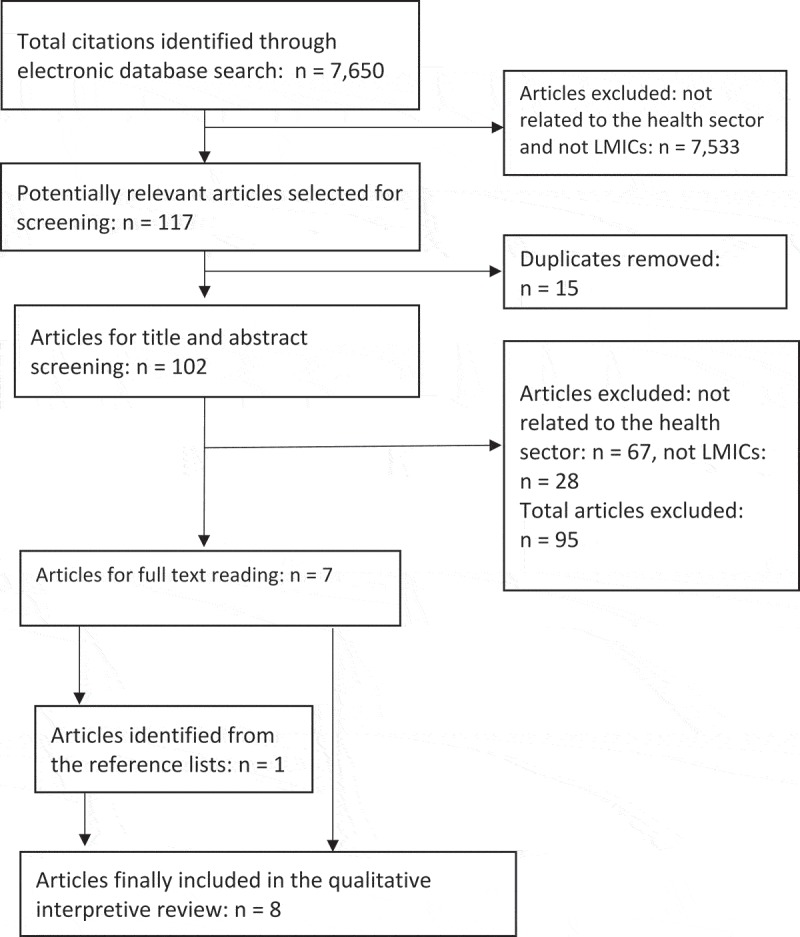
Search flow diagram.

The initial literature search generated 7650 articles. The majority of the articles were excluded because they were unrelated to the health sector and they had a focus in high-income countries (HICs). One hundred and seventeen articles were thought to be potentially relevant. Following the removal of duplicates, 102 articles remained and their titles and abstracts were screened against the inclusion criteria. Only seven articles were retrieved for full text reading with one additional article retrieved from searching the reference lists of these articles, generating a total of eight articles. This process is outlined in Figure 2 with reasons for article exclusion indicated at each stage.

### Characteristics of the literature

The characteristics of the articles included in the review vary in terms of type of health sector reform considered, country and organisations of focus and methodology used, as outlined in Table 2. The organisations of focus in most of the papers were public health sector organisations (n = 7) involving either the public district health system or the national ministry of health.

**Table 2.** Characteristics of the literature.

Type of health sector reform	Country and organisations of focus	Methodology
Decentralisation (n = 6)	Brazil (n = 2); Public sector district health system	Ethnographic studies (n = 2)
	Ghana (n = 3); Public sector district health system	Qualitative studies (n = 3)
	Uganda (n = 1); National Ministry of Health	Case study (n = 1)
Comprehensive health sector reform to strengthen primary health care (n = 1)	Nigeria; 4 hospitals: Three public sector hospitals and one not-for- profit mission hospital	Quantitative study
Public-private partnerships between the State and Civil Society Organisations (n = 1)	India; Partnership between two civil society organisations and the public state	Multi-site ethnographic studies

Three of the six articles on decentralisation have the same first author and were carried out in Ghana but in different districts [37–39]. All articles offer insights into the influence of organisational culture over the implementation of the health sector reforms, with the articles on decentralisation offering more insight not only because they constituted the majority of the articles (n = 6), but also because they used case studies and qualitative methods to provide a rich description of the implementation experience and context. However, only one of the eight articles included in the review explicitly set out to study organisational culture and this study used a quantitative survey to assess organisational culture across a range of hospitals [40]. A full list and brief overview of these articles is provided in a table in Supplementary material 2.

### Description of findings

The following section reports on the practices and values that were inductively identified from the retrieved articles as influencing the implementation of health sector reforms primarily within public health sector settings. These practices and values were identified from the interview reports, survey responses and observations (e.g. of district meetings) reported in the papers, as well as authorial judgements. These practices include communication, management styles, participation in decision-making and commitment. They do not, however, occur in isolation and interactions, and overlaps can be seen across them. Management styles and participation in decision-making showed marked overlap and are therefore presented under one finding. This section concludes with an interpretive synthesis and summary of the findings.

### Communication practices

The majority of papers demonstrated weaknesses in communication practices as an influence over health reform implementation mostly, but not exclusively, in public health sector organisations [37–41]. These weaknesses convey implicit and explicit values judgements of how communication should be carried out as reported by participants or as interpreted and judged by the authors.

### Awareness, clarity and adequacy of information

In Ghana, some public health workers and external stakeholders in the district health system were not aware of the decentralisation policy and its aims which undermined the support needed to implement the reform:

‘I am not aware of the decentralisation programme … I don’t also know the aims for decentralisation’ [38].

Public health workers and other stakeholders also reported receiving little or no information on major reforms from public district health system managers. This further undermined health workers’ knowledge of the reform and its objectives and slowed the implementation of the reform [37]. Interestingly, senior health managers held the opinion that members of the staff should only receive information that was relevant to them:

‘Staff are not supposed to be given all the information, only information that concerns them or what they need to know is made available to them’ [37].

The lack of awareness and inadequate information resulted in dysfunctional interactions between the district health team, public health workers and external stakeholders [37–39]. It also led to an attachment to the old values and systems of doing things which limited the implementation of the decentralisation policy in Ghana:

[W]eak communication and information sharing contributed to the limited understanding of reform. This constrained reform implementation because, instead of opening up to the challenges and opportunities brought about by the reform, health workforces continued to hold onto the old value system and its style of service management in an era of change [37].

In Nigeria, public health workers and those from one mission hospital reported that information was not openly shared in their hospitals. This was perceived as an area of weakness that required strengthening in order to support the implementation of the comprehensive health sector reform [40].

### Timeliness of information and feedback

Public health workers and district stakeholders across Ghana’s district health system reported that district health managers did not provide information in time:

The only problem was that mostly the district assembly received the information late [37].

We only hear of the programmes either on radio or when the programme is finished [37].

Public health workers also complained about non-response and delays in receiving feedback from their managers. In contrast, information sharing and provision of feedback among senior public health managers was perceived to occur frequently:

‘District health directors and managers communicate and share relevant information with senior managers, and they do so frequently; and, they do regular follow-up for feedback, either by telephone or written note’ [37].

According to Sakyi [37], delays and lack of feedback arose from heavy dependence on the top-down style of communication which led to centralisation of information among the public health managers. This prevented public health workers and external stakeholders from learning about the reform process which subsequently constrained the implementation of the decentralisation policy.

### Effectiveness of forms of communication

In Ghana, reports by public health workers and district stakeholders indicate that the usual forms of government communication, such as circulars, letters, memos and reports, were not being used effectively to share information on management decisions which limited their knowledge of the decentralisation policies [37,38]:

‘Although we know that there is a decentralised policy in the system, we have not officially been informed and had not got any written document about it so we are not very clear about its content’ [38].

In Uganda, the use of circulars and written communication, as opposed to face-to-face communication, were seen as the cause of poor communication between the Ministry of Health and the district health system. This resulted in poor support for the restructuring process required for the decentralisation policies at the district level.

‘It seems obvious that in Uganda, circulars and written communication in general may not suffice as carrying contexts. Important processes such as the critical face-to-face relationship, the “co-presence” in space and time, need to be directly and clearly established’ [41].

In India, the government and one Civil Society Organisation (CSO) used posters to convey messages of equal participation in decision-making as part of decentralising health care planning to the local level. However, the effectiveness of this communication was undermined by the broader social and gender hierarchies that limited participation in these councils [42].

### Management styles

Three kinds of management styles were described in the reviewed articles: authoritarian, participative and consultative [37–39,41–44]. These management styles had different influences on the implementation of health sector reforms, as seen below.

### Authoritarian management

Authoritarian management was often characterised by hierarchy and centralisation of power and communication. In Ghana, public sector hierarchical structures were seen as barriers to the decentralisation policy because they negatively affected the attitude, behaviour and interactions of different actors in the district health system:

‘The way the structures are put up here does not help; the policy is not well practised here because it is a one person’s administration’ [38].

Atkinson et al. [44] examined public health system decentralisation in three districts (one rural, one urban and one metropolitan) in Brazil. They observed that decision-making power in the rural district was centralised to the district prefect – a political figure – who never consulted health staff or members of the health council. As a result, the health secretariat and staff lacked autonomy and voice in decision-making, which led to the poor implementation of the reform policy [44].

In India, members of the Civil Society Organisations (CSOs) who had partnered with the State of India to promote rural heath reported that the State dominated the partnership. This dominance highlighted the bureaucratic and hierarchical nature of the government, which led to asymmetry in the State–CSOs partnership with varying forms of conflict that challenged and threatened the sustainability of the State–CSOs partnerships. The dominance also stifled the autonomy of the CSOs and limited the effectiveness of the CSO–government partnership [42]:

‘When we work with you we have lost the liberty. Because we think according to you, we plan according to you, we get our salary according to you. So that is the reason why we are not doing well’ [42].

Perceptions of loss of power by some of the actors at the regional and central public administration in Ghana were judged as barriers to the implementation of the decentralisation policy [38]. In Uganda, the paternalistic attitude of Ministry of Health staff towards the district health system and the attachment to the traditional way of managing programmes within the Ministry of Health – in the face of the restructuring process and decentralisation – were seen as ‘bureaucratic resistance to decentralisation’ [41]. This resulted in poor ownership of the restructuring and decentralisation policies by public officials at the district level.

Authoritarian management was also inferred from reports of hierarchical reporting lines among actors in the health system. For example, managers in Ghana’s Sekyere district health system were required to seek approval from the regional administration prior to making any decisions:

In the event of any needed change, health directors had to seek prior permission and must wait until approval is granted from regional or headquarters before any action could be taken [39].

[T]he top would have to come in before we are able to take decisions [38].

Reporting lines between Ghana’s district health system and the regional departments formed barriers to decision-making and implementation of the decentralisation policy. In addition, conflict over reporting lines between the district assembly officials and district health officials in Ghana’s Sekyere district undermined cooperation and collaboration, which further weakened implementation of the decentralisation policy in this district [39].

### Participative management

In participative management, public health managers encouraged the participation of their own health workers and external stakeholders in the reform process. In Brazil’s metropolitan district, the district health secretary’s style of management was participative because the secretary encouraged the participation of health workers in decision-making. However, the secretary did not engage the district health council because of the assumption that the district health council (made up of both health workers and lay members of the community) was a bureaucratic intervention. As a result, the council no longer convened, which slowed the implementation of the decentralisation policy [44]. In Nigeria, hospital managers encouraged team work and participation of the staff in planning for the health sector reforms. This style of management was seen as a supportive element of culture for the reform process [40]. On the other hand, public health managers in Ghana did not did encourage external stakeholder (district assemblies, non-governmental organisations [NGOs] and private providers) participation in planning and decision-making. This undermined the stakeholders’ knowledge of the ongoing decentralisation reforms and weakened the implementation of the policy [37,39]. Public health workers and external stakeholders in Ghana’s district health system considered participation in decision-making essential for the successful implementation of the decentralisation policy [37–39].

### Consultative management

Interview reports from external stakeholders in Ghana indicate that it was an uncommon practice for the district health managers to consult health workers and external stakeholders on management issues including major reforms:

‘The district management does not give information, or consult us about any health management issues or major health decision’ [37].

The poor consultation and involvement of stakeholders resulted in their exclusion from the decentralisation reform process, which weakened the support needed for the implementation of the decentralisation reform [37]:

‘Professional associations were not informed or effectively absorbed into the health reform programme’ [37].

In Brazil’s urban district health system, the district health secretary employed a consultative style of management. As a result, the decentralisation policy was judged as better implemented in the urban district than in the rural and metropolitan districts [44].

### Commitment

Commitment of district health managers to the districts and to the reforms was judged as an important aspect of the social organisation that influenced the implementation of the decentralisation policy in Brazil [43,44]. For instance, health managers in Brazil’s urban district health system were observed to be the most committed to the reform objectives in terms of the language used and adherence to the procedures outlined in the reforms when compared to the managers and health workers in the rural and metropolitan districts. Consequently, decentralisation policy was better implemented in the urban district than in the metropolitan and rural districts [43,44]. In Ghana’s Nkwanta district, health managers reported poor commitment to and lack of ownership of the decentralisation policy because they felt that the headquarters largely imposed the reforms on them. Poor commitment and lack of ownership created barriers to the decentralisation policy [39].

In Nigeria, inferences on commitment were made at both the hospital level and the managers’ level. At hospital level, commitment was inferred from the health workers’ report that both public and mission hospital activities and images were consistent with the objectives of the reforms. These hospitals were also seen to have the capacity to innovate towards the reforms. At managerial level, health managers from both public and mission hospitals were committed to representing the interests of the hospitals to external stakeholders and to steering the organisation towards achieving the objectives of the health sector reforms. These aspects of organisational culture were strong enough to support the implementation of the health sector reforms [40].

### Influence of the wider social and political context

The influence of political culture on the implementation of the decentralisation policy was judged as particularly marked in Brazil’s rural district compared to Brazil’s urban and metropolitan districts [44] such that the decentralisation reform had little impact on increasing local voice and autonomy [43,44]. Authorial observations of Brazil’s rural district showed that the district prefect – a political figure – retained all the decision-making power, thereby disempowering the district health secretary and limiting the participation of the health workers in decision-making. In addition, authorial judgments indicate that the disposition and behaviour of the district health secretary towards the health sector reforms in Brazil’s district health systems mirrored what was valued by the political leaders including the district prefect. In this regard, the prevailing political culture in Brazil’s rural district hindered the implementation of the decentralisation [43,44]. According to Atkinson et al. [44], political culture and social organisation had the potential to negatively influence the implementation of the decentralisation policy across Brazil:

‘The extent to which aspects of social organisation and political culture enable or hinder implementation indicates a mixed influence but one which is sufficiently negative.’ .

In Ghana’s Nkwanta district, political interference by those in authority formed a barrier to the implementation of the decentralisation policy [38], while in India efforts by the Indian government and the CSOs to increase local participation in decision-making in the village health councils as part of the decentralisation of health planning were limited by the wider social and gender hierarchies [42].

### Synthesis of the findings

**Figure 3.**
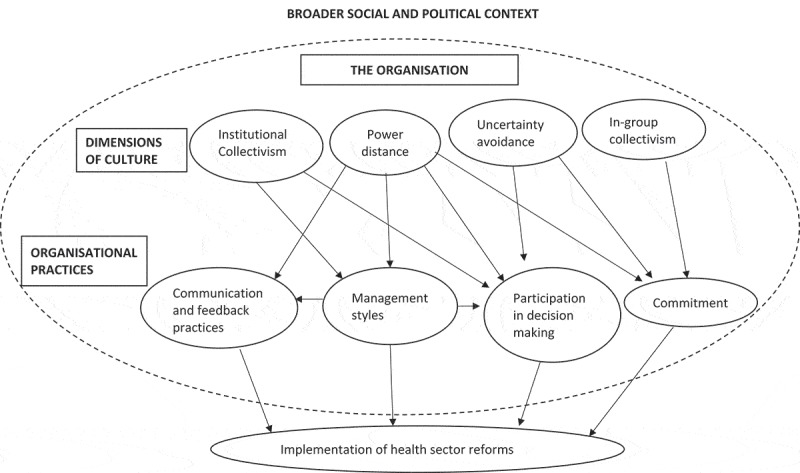
Framework of the relationship between dimensions of organisational culture, organisational practices and implementation of health sector reforms. Adapted from Jaakko et al. [46]. The boundary of the organisation is represented by a dotted line to show the influence of the wider political and social context.

The study of culture within organisations is largely interpretive and founded on the notion that the behaviour and actions of the members are influenced by rules, orders, incentives and ‘common frames of reference’ [45]. In the previous section, we presented the range of practices and values inductively identified from the papers that point to the influence of public sector organisational culture over reform implementation. In this section we seek to deepen understanding of these influences through synthesis and further interpretation, drawing on four of the organisational cultural dimensions proposed by House et al. [22]: power distance, in-group collectivism, uncertainty avoidance and institutional collectivism. These four dimensions are those most clearly visible in the identified organisational practices. As summarised in Figure 3, moreover, the organisational practices identified inductively seem to mediate the influence of these particular and interacting dimensions of organisational culture over the implementation of health system reforms, and all are themselves influenced by the broader sociopolitical context.

*Power distance* is characterised by varying concentrations and distributions of power across the health systems, with varying impacts on organisational practices and implementation of reforms. For instance, the presence of a large power distance in the district health system – characterised by centralisation of power to the district managers – not only disempowered the junior managers, but also limited the autonomy, local voice and participation in decision-making by health workers and external stakeholders as seen across Brazil’s and Ghana’s case studies. This weakened the implementation of the decentralisation policy in both countries [37–39,43,44]. This large power distance also negatively affected the commitment of managers and health workers in Brazil’s rural district [43,44] and in Ghana’s Sekyere district [39], which further weakened the implementation of the decentralisation policy. In Ghana, the presence of a large power distance led to the concentration of communication among the senior public health managers and to the dependence on top-down style of communication. This resulted in poor communication and feedback practices between the managers and health workers, which led to poor implementation of the decentralisation reform at the district level [37–39]. In India, the large power distance between the government and the CSOs limited the autonomy of the CSOs and threatened the sustainability of the partnership [42]. On the other hand, the presence of a small power distance was associated with participative and consultative styles of management as well as increased participation of health workers and stakeholders in decision-making, as seen in Brazil’s urban district, leading to better implementation of the reform compared to the rural and metropolitan districts [44]. The above case studies therefore suggest that the extent of power distance within the health system can shape the implementation of the reforms through its influence on management styles, participation in decision-making, communication and commitment.

*High institutional collectivism* can be inferred from local health systems that valued team work and collective action from both the members of the organisation and the external stakeholders. For instance, Brazil’s urban health system valued collective consultation and participation of health workers and stakeholders in decision-making. The district health secretary consulted and engaged the stakeholders in regular health council meetings. This created an enabling environment for the implementation of the decentralisation policy, which proceeded with fewer challenges compared to the metropolitan and rural districts [43,44]. However, local health systems with low institutional collectivism did not encourage collective action in decision-making for the reform process from either their members or external stakeholders, as seen in Brazil’s rural district [44], in Ghana’s district health system [37–39] and in Uganda [41]. Low collectivism, therefore, undermined both intra-organisational and inter-organisational support for the reforms, which resulted in the poor implementation of the health sector reforms in these studies. It can therefore be inferred and interpreted from these studies that the extent to which the organisation values institutional collectivism will influence the management style as well as level of health worker or stakeholder participation in decision-making.

*High in-group collectivism* was expressed by managers in Brazil’s urban district who showed more commitment to the district and to the decentralisation reform than the managers in the rural and metropolitan district. This commitment was judged to lead to better implementation of the reform in the urban district [43,44]. High in-group collectivism can also be inferred in the Nigerian study, where health workers expressed confidence in the capacity of their managers to steer the organisation towards achieving the reform objectives by aligning organisational activities to the reform objectives, which supported the reform process [40].

*Uncertainty avoidance* was inferred from Ghana’s district health system where, despite health workers’ perception of needed change, no decisions could be made without the approval of senior managers. The heavy dependence on rules and approval to guide decision-making despite needed change is suggestive of high uncertainty avoidance. Unfortunately, this slowed decision-making for the implementation of the reforms [38]. In Uganda, the attachment to the traditional practice of managing vertical programmes by Ministry of Health officials in the face of decentralisation and restructuring underscores organisational rigidity to change and hence high uncertainty avoidance, which also limited the implementation of these policies [41]. On the other hand, an organisation’s capacity to innovate, as reported by the health workers in Nigeria, is suggestive of low uncertainty avoidance, which was seen to support implementation of the reforms [40].

Beyond these dimensions of organisational culture, the reviewed literature also provided evidence of the influence of the *wider social and political culture* on organisational practices and subsequently on the implementation of the health sector reforms. This influence was particularly felt in Brazil’s rural district health system where the prevailing political culture and attitudes of political leaders influenced the management styles and extent of participation of health managers and health workers in decision-making [43,44]. Political culture in Brazil and political interference in Ghana’s Nkwanta district [38] limited the implementation of the decentralisation policy. In India, social and gender hierarchies limited participation in decision-making, which undermined the efforts to decentralise health planning under the State–CSOs partnership [42].

## Discussion

There has been growing interest in the notion of organisational culture and its potential influence in the health sector in HICs. However, organisational culture has been little examined in health system studies in LMICs. This paper, therefore, presents a review of empirical literature with two aims: (a) to identify and synthesise findings about organisational culture and its influence on the implementation of health sector reforms in LMICs, and (b) to provide analytic generalisations that can inform health policy and systems research. The retrieval of only a few papers can be seen as a limit of this synthesis; however, analytic generalisation is possible and it provides the following insights.

Using thematic analysis, this review identified four organisational practices that influenced the implementation of health sector reforms in public health sector organisations across different country settings: communication, management styles, participation in decision-making, and commitment. To deepen understanding of these organisational practices as dimensions of organisational culture, they were further synthesised and interpreted using the dimensions of power distance, in-group collectivism, institutional collectivism and uncertainty avoidance [22].

Articulating the nature of the influence of organisational culture on the implementation of health sector reforms was largely based on judgements and new insights beyond those of the primary studies, in keeping with the aim of an interpretive synthesis [25]. The interpretations arrived at in this review suggest that: (a) power distance impacts on communication, management styles, commitment and participation in decision-making; (b) institutional collectivism impacts on management practices and participation in decision-making; (c) uncertainty avoidance impacts on decision-making and commitment; and (d) in-group collectivism impacts on commitment, as summarised in Figure 3 above.

The multiple linkages between these cultural dimensions and organisational practices highlight the complexity of the notion of culture within organisations. Nevertheless, the interpretations arrived at in this synthesis can be supported by wider literature. Power distance is expected in any society or organisation, with some showing more inequality than others [47]. As seen in this review, power distance varied across the district health systems in different countries, as seen across the three district health systems in Brazil. The influence of power distance on the style of management and participation in decision-making is not peculiar to the health sector. A large multi-country study on the influence of culture on managers’ behaviour across different continents, including Africa, showed that in a hierarchical culture managers tended to rely on rules, procedures and their superiors during decision-making and less on their subordinates [48]. Similarly, the influence of power distance and collectivism on organisational practices appeared to overlap, leading to different forms of management styles (authoritarian, consultative or participatory) and participation in decision-making. Interestingly, both power distance and collectivism have also been shown to correlate in various country settings, leading to various forms of participatory decision-making depending on the extent to which both cultural dimensions are valued and practised within the organisation [49]. The effect of the broader social and political culture on organisational culture and implementation of health sector reforms is supported by Gilson and Erasmus [50], who recognised that organisations are embedded within the wider society and can therefore be influenced by societal values.

This review has the following implications for health policy and systems research. First, given the dearth of literature, it underscores the need for more empirical studies on organisational culture and its influence on reform implementation in the health sector. It is possible that these studies may generate new insights on how different dimensions of organisational culture, values and practices influence changes in the health sector which may be useful for health system development. As highlighted in Figure 3, such understanding must also consider the influence of the broader sociopolitical context. Second, the framework presented in Figure 3 provides a useful starting point for future researchers to test and build the knowledge base on organisational culture and reforms in the health sector. This framework may also support further cross-paper or cross-context analysis in interpretive synthesis work and qualitative empirical research. Third, future researchers can also build on this interpretive synthesis – for example, by considering unpublished literature and literature from HICs, as well as by expanding and translating the literature search strategy to other data bases accessible to them. This would address a limitation of this review which excluded articles published in languages other than English and unpublished literature. Lastly, the broad and inclusive scope of organisational culture makes its interpretation difficult. We therefore recommend that future researchers work in teams when studying and analysing organisational culture, in order to generate a richer analysis drawing on the different perspectives and experiences of the research team.

With regard to the implications for health managers and policymakers, the findings of this review suggest the value of identifying dimensions of organisational culture which can influence the implementation of health sector reforms indirectly by influencing organisational practices. Due to the limited number of articles reviewed, no conclusions can be made on which dimensions of organisational culture provide the most influence on the implementation of health sector reforms – although it can be inferred that power distance was an important influence on organisational practices. Understanding culture can, then, facilitate the development and negotiation of ‘mutually agreeable approaches to conflict resolution, problem solving, decision making, and management practices’ [22], all of which characterised the implementation of the reforms across the different settings in the reviewed literature. It is important that improvement strategies are adapted to the local context [47] as what works in one context may not necessarily work in another. The importance of organisational culture in the health sector cannot be overemphasised.

## Conclusion

This interpretive synthesis suggests the potential influence of dimensions of organisational culture such as power distance, in-group collectivism, institutional collectivism and uncertainty avoidance on the implementation of health sector reforms. Their influence appears to be mediated through organisational practices such as management styles, participation in decision-making, communication and commitment. However, the analytic generalisations drawn from this synthesis are limited by the few papers retrieved. More empirical research on organisational culture in LMIC health systems is needed in order to deepen understanding of its influence on health reform implementation and health system development.

## Paper context

The World Development Report of 1993 led to the introduction of health sector reforms with the aim of improving efficiency, effectiveness and responsiveness of health systems globally. The implementation of these reforms has, however, resulted in limited improvement. One plausible explanation for this is the inattention to organisational culture, which is thought to be more likely to impede change. This review uses existing empirical literature to explore evidence on organisational culture and its influence on the implementation of health sector reforms in low- and middle-income countries.
