# Supplementary material for: Influence of organisational culture on the implementation of health sector reforms in low- and middle-income countries: a qualitative interpretive review
Source: Glob Health Action. 2018 May 11;11(1):1462579. doi: 10.1080/16549716.2018.1462579 (PMC5954479; doi:10.1080/16549716.2018.1462579)
Supplement: Supplementary material [file ZGHA_A_1462579_SM0646.zip › Supplementary material 1_literature search strategy.docx]

# S1 Appendix. Full literature search strategy

**PUBMED SEARCH STRATEGY (24)**

(((((((comprehensive[All Fields] AND ("primary health care"[MeSH Terms] OR ("primary"[All Fields] AND "health"[All Fields] AND "care"[All Fields]) OR "primary health care"[All Fields])) OR (("public-private sector partnerships"[MeSH Terms] OR ("public-private"[All Fields] AND "sector"[All Fields] AND "partnerships"[All Fields]) OR "public-private sector partnerships"[All Fields] OR ("public"[All Fields] AND "private"[All Fields] AND "partnership"[All Fields]) OR "public private partnership"[All Fields]) AND ("2000/01/01"[PDAT] : "3000/12/31"[PDAT]))) OR ("Public-Private Sector Partnerships"[Mesh] AND ("2000/01/01"[PDAT] : "3000/12/31"[PDAT])) AND (Filters[All Fields] AND (("publishing"[MeSH Terms] OR "publishing"[All Fields] OR "publication"[All Fields] OR "publications"[MeSH Terms] OR "publications"[All Fields]) AND date[All Fields] AND 2000/01/01[All Fields])) OR ((outsource[All Fields] OR outsource'[All Fields] OR outsourceability[All Fields] OR ("outsourced services"[MeSH Terms] OR ("outsourced"[All Fields] AND "services"[All Fields]) OR "outsourced services"[All Fields] OR "outsourced"[All Fields]) OR outsourced'[All Fields] OR outsourcee[All Fields] OR outsourcer[All Fields] OR outsourcer's[All Fields] OR outsourcers[All Fields] OR outsources[All Fields] OR ("outsourced services"[MeSH Terms] OR ("outsourced"[All Fields] AND "services"[All Fields]) OR "outsourced services"[All Fields] OR "outsourcing"[All Fields]) OR outsourcing'[All Fields] OR outsourcing's[All Fields]) OR "Outsourced Services"[Mesh]) AND ("Primary Health Care"[Mesh] OR ("primary health care"[MeSH Terms] OR ("primary"[All Fields] AND "health"[All Fields] AND "care"[All Fields]) OR "primary health care"[All Fields])) AND ("Politics"[Mesh] AND ("2000/01/01"[PDAT] : "3000/12/31"[PDAT]))) OR (("politics"[MeSH Terms] OR "politics"[All Fields] OR "decentralization"[All Fields]) AND ("2000/01/01"[PDAT] : "3000/12/31"[PDAT]))) OR (decentralisation[All Fields] AND ("2000/01/01"[PDAT] : "3000/12/31"[PDAT]))) OR ((((((((((((((((("health"[MeSH Terms] OR "health"[All Fields]) AND sector[All Fields] AND reform[All Fields]) OR (("health"[MeSH Terms] OR "health"[All Fields]) AND sector[All Fields] AND reforms[All Fields])) OR "Health Care Reform"[Mesh]) OR (health care reform[All Fields] OR health care reform's[All Fields] OR health care reformation[All Fields] OR health care reformers[All Fields] OR health care reforms[All Fields])) OR "Health Policy"[Mesh]) OR (health police[All Fields] OR health polices[All Fields] OR health policies[All Fields] OR health policing[All Fields] OR health policy[All Fields] OR health policyand[All Fields] OR health policymaker[All Fields] OR health policymakers[All Fields] OR health policymaking[All Fields])) OR (("health"[MeSH Terms] OR "health"[All Fields]) AND system[All Fields] AND strengthening[All Fields] AND ("Intervention (Amstelveen)"[Journal] OR "intervention"[All Fields] OR "Interv Sch Clin"[Journal] OR "intervention"[All Fields]))) OR (("health"[MeSH Terms] OR "health"[All Fields]) AND system[All Fields] AND strengthening[All Fields] AND interventions[All Fields])) OR (("Universal"[Supplementary Concept] OR "Universal"[All Fields] OR "universal"[All Fields]) AND ("health"[MeSH Terms] OR "health"[All Fields]) AND ("AHIP Cover"[Journal] OR "coverage"[All Fields]))) OR "user fee removal"[All Fields]) OR "Reimbursement, Incentive"[Mesh]) OR "pay for performance"[All Fields]) OR "health sector strategy"[All Fields]) OR (health sector strategic[All Fields] OR health sector strategies[All Fields] OR health sector strategy[All Fields])) OR "health system reform"[All Fields]) OR "health reform"[All Fields] OR (user[All Fields] AND ("economics"[Subheading] OR "economics"[All Fields] OR "fees"[All Fields] OR "fees and charges"[MeSH Terms] OR ("fees"[All Fields] AND "charges"[All Fields]) OR "fees and charges"[All Fields]))) OR (performance[All Fields] AND based[All Fields] AND ("economics"[Subheading] OR "economics"[All Fields] OR "financing"[All Fields] OR "economics"[MeSH Terms] OR "financing"[All Fields]))) OR "performance based financing"[All Fields]) AND ((((institutional[All Fields] AND ("ethnology"[Subheading] OR "ethnology"[All Fields] OR "culture"[All Fields] OR "culture"[MeSH Terms]) AND Filters[All Fields]) AND (("publishing"[MeSH Terms] OR "publishing"[All Fields] OR "publication"[All Fields] OR "publications"[MeSH Terms] OR "publications"[All Fields]) AND date[All Fields] AND 2000/01/01[All Fields])) OR "Organizational Culture"[Mesh] OR (organizational culture[Title/Abstract] OR organizational cultures[Title/Abstract])) OR (organisational culture[All Fields] OR organisational cultures[All Fields])) AND ("Health Plan Implementation"[Mesh] OR implementation[All Fields]) AND (((("developing countries"[MeSH Terms:noexp] OR "africa"[MeSH Terms:noexp] OR "africa, northern"[MeSH Terms:noexp] OR "africa south of the sahara"[MeSH Terms:noexp] OR "africa, central"[MeSH Terms:noexp] OR "africa, eastern"[MeSH Terms:noexp] OR "africa, southern"[MeSH Terms:noexp] OR "africa, western"[MeSH Terms:noexp] OR "asia"[MeSH Terms:noexp] OR "asia, central"[MeSH Terms:noexp] OR "asia, southeastern"[MeSH Terms:noexp] OR "asia, western"[MeSH Terms:noexp] OR "caribbean region"[MeSH Terms:noexp] OR "west indies"[MeSH Terms:noexp] OR "south america"[MeSH Terms:noexp] OR "latin america"[MeSH Terms:noexp] OR "central america"[MeSH Terms:noexp] OR "afghanistan"[MeSH Terms:noexp] OR "albania"[MeSH Terms:noexp] OR "algeria"[MeSH Terms:noexp] OR "american samoa"[MeSH Terms:noexp] OR "angola"[MeSH Terms:noexp] OR "Antigua and Barbuda"[Mesh:noexp] OR "argentina"[MeSH Terms:noexp] OR "armenia"[MeSH Terms:noexp] OR "azerbaijan"[MeSH Terms:noexp] OR "bahrain"[MeSH Terms:noexp] OR "bangladesh"[MeSH Terms:noexp] OR "barbados"[MeSH Terms:noexp] OR "benin"[MeSH Terms:noexp] OR "republic of belarus"[MeSH Terms:noexp] OR "belize"[MeSH Terms:noexp] OR "bhutan"[MeSH Terms:noexp] OR "bolivia"[MeSH Terms:noexp] OR "bosnia and herzegovina"[MeSH Terms:noexp] OR "botswana"[MeSH Terms:noexp] OR "brazil"[MeSH Terms:noexp] OR "bulgaria"[MeSH Terms:noexp] OR "burkina faso"[MeSH Terms:noexp] OR "burundi"[MeSH Terms:noexp] OR "cambodia"[MeSH Terms:noexp] OR "cameroon"[MeSH Terms:noexp] OR "cape verde"[MeSH Terms:noexp] OR "central african republic"[MeSH Terms:noexp] OR "chad"[MeSH Terms:noexp] OR "chile"[MeSH Terms:noexp] OR "china"[MeSH Terms:noexp] OR "colombia"[MeSH Terms:noexp] OR "comoros"[MeSH Terms:noexp] OR "congo"[MeSH Terms:noexp] OR "costa rica"[MeSH Terms:noexp] OR "cote d'ivoire"[MeSH Terms:noexp] OR "croatia"[MeSH Terms:noexp] OR "cuba"[MeSH Terms:noexp] OR "cyprus"[MeSH Terms:noexp] OR "czechoslovakia"[MeSH Terms:noexp] OR "czech republic"[MeSH Terms:noexp] OR "slovakia"[MeSH Terms:noexp] OR "djibouti"[MeSH Terms:noexp] OR "Democratic Republic of the Congo"[Mesh:noexp] OR "dominica"[MeSH Terms:noexp] OR "dominican republic"[MeSH Terms:noexp] OR "timor-leste"[MeSH Terms:noexp] OR "ecuador"[MeSH Terms:noexp] OR "egypt"[MeSH Terms:noexp] OR "el salvador"[MeSH Terms:noexp] OR "eritrea"[MeSH Terms:noexp] OR "estonia"[MeSH Terms:noexp] OR "ethiopia"[MeSH Terms:noexp] OR "fiji"[MeSH Terms:noexp] OR "gabon"[MeSH Terms:noexp] OR "gambia"[MeSH Terms:noexp] OR "Georgia (Republic)"[Mesh:noexp] OR "ghana"[MeSH Terms:noexp] OR "greece"[MeSH Terms:noexp] OR "grenada"[MeSH Terms:noexp] OR "guatemala"[MeSH Terms:noexp] OR "guinea"[MeSH Terms:noexp] OR "guinea-bissau"[MeSH Terms:noexp] OR "guam"[MeSH Terms:noexp] OR "guyana"[MeSH Terms:noexp] OR "haiti"[MeSH Terms:noexp] OR "honduras"[MeSH Terms:noexp] OR "hungary"[MeSH Terms:noexp] OR "india"[MeSH Terms:noexp] OR "indonesia"[MeSH Terms:noexp] OR "iran"[MeSH Terms:noexp] OR "iraq"[MeSH Terms:noexp] OR "jamaica"[MeSH Terms:noexp] OR "jordan"[MeSH Terms:noexp] OR "kazakhstan"[MeSH Terms:noexp] OR "kenya"[MeSH Terms:noexp] OR "korea"[MeSH Terms:noexp] OR "kosovo"[MeSH Terms:noexp] OR "kyrgyzstan"[MeSH Terms:noexp] OR "laos"[MeSH Terms:noexp] OR "latvia"[MeSH Terms:noexp] OR "lebanon"[MeSH Terms:noexp] OR "lesotho"[MeSH Terms:noexp] OR "liberia"[MeSH Terms:noexp] OR "libya"[MeSH Terms:noexp] OR "lithuania"[MeSH Terms:noexp] OR "macedonia (republic)"[MeSH Terms:noexp] OR "madagascar"[MeSH Terms:noexp] OR "malaysia"[MeSH Terms:noexp] OR "malawi"[MeSH Terms:noexp] OR "mali"[MeSH Terms:noexp] OR "malta"[MeSH Terms:noexp] OR "mauritania"[MeSH Terms:noexp] OR "mauritius"[MeSH Terms:noexp] OR "mexico"[MeSH Terms:noexp] OR "micronesia"[MeSH Terms:noexp] OR "middle east"[MeSH Terms:noexp] OR "moldova"[MeSH Terms:noexp] OR "mongolia"[MeSH Terms:noexp] OR "montenegro"[MeSH Terms:noexp] OR "morocco"[MeSH Terms:noexp] OR "mozambique"[MeSH Terms:noexp] OR "myanmar"[MeSH Terms:noexp] OR "namibia"[MeSH Terms:noexp] OR "nepal"[MeSH Terms:noexp] OR "netherlands antilles"[MeSH Terms:noexp] OR "new caledonia"[MeSH Terms:noexp] OR "nicaragua"[MeSH Terms:noexp] OR "niger"[MeSH Terms:noexp] OR "nigeria"[MeSH Terms:noexp] OR "oman"[MeSH Terms:noexp] OR "pakistan"[MeSH Terms:noexp] OR "palau"[MeSH Terms:noexp] OR "panama"[MeSH Terms:noexp] OR "papua new guinea"[MeSH Terms:noexp] OR "paraguay"[MeSH Terms:noexp] OR "peru"[MeSH Terms:noexp] OR "philippines"[MeSH Terms:noexp] OR "poland"[MeSH Terms:noexp] OR "portugal"[MeSH Terms:noexp] OR "puerto rico"[MeSH Terms:noexp] OR "romania"[MeSH Terms:noexp] OR "russia"[MeSH Terms:noexp] OR "Russia (Pre-1917)"[Mesh:noexp] OR "rwanda"[MeSH Terms:noexp] OR "Saint Kitts and Nevis"[Mesh:noexp] OR "saint lucia"[MeSH Terms:noexp] OR "Saint Vincent and the Grenadines"[Mesh:noexp] OR "samoa"[MeSH Terms:noexp] OR "saudi arabia"[MeSH Terms:noexp] OR "senegal"[MeSH Terms:noexp] OR "serbia"[MeSH Terms:noexp] OR "montenegro"[MeSH Terms:noexp] OR "seychelles"[MeSH Terms:noexp] OR "sierra leone"[MeSH Terms:noexp] OR "slovenia"[MeSH Terms:noexp] OR "sri lanka"[MeSH Terms:noexp] OR "somalia"[MeSH Terms:noexp] OR "south africa"[MeSH Terms:noexp] OR "sudan"[MeSH Terms:noexp] OR "suriname"[MeSH Terms:noexp] OR "swaziland"[MeSH Terms:noexp] OR "syria"[MeSH Terms:noexp] OR "tajikistan"[MeSH Terms:noexp] OR "tanzania"[MeSH Terms:noexp] OR "thailand"[MeSH Terms:noexp] OR "togo"[MeSH Terms:noexp] OR "tonga"[MeSH Terms:noexp] OR "Trinidad and Tobago"[Mesh:noexp] OR "tunisia"[MeSH Terms:noexp] OR "turkey"[MeSH Terms:noexp] OR "turkmenistan"[MeSH Terms:noexp] OR "uganda"[MeSH Terms:noexp] OR "ukraine"[MeSH Terms:noexp] OR "uruguay"[MeSH Terms:noexp] OR "ussr"[MeSH Terms:noexp] OR "uzbekistan"[MeSH Terms:noexp] OR "vanuatu"[MeSH Terms:noexp] OR "venezuela"[MeSH Terms:noexp] OR "vietnam"[MeSH Terms:noexp] OR "yemen"[MeSH Terms:noexp] OR "yugoslavia"[MeSH Terms:noexp] OR "zambia"[MeSH Terms:noexp] OR "zimbabwe"[MeSH Terms:noexp]) OR (Macedonia[tw] OR Madagascar[tw] OR Malagasy Republic[tw] OR Malaysia[tw] OR Malaya[tw] OR Malay[tw] OR Sabah[tw] OR Sarawak[tw] OR Malawi[tw] OR Nyasaland[tw] OR Mali[tw] OR Malta[tw] OR Marshall Islands[tw] OR Mauritania[tw] OR Mauritius[tw] OR Agalega Islands[tw] OR Mexico[tw] OR Micronesia[tw] OR Middle East[tw] OR Moldova[tw] OR Moldovia[tw] OR Moldovian[tw] OR Mongolia[tw] OR Montenegro[tw] OR Morocco[tw] OR Ifni[tw] OR Mozambique[tw] OR Myanmar[tw] OR Myanma[tw] OR Burma[tw] OR Namibia[tw] OR Nepal[tw] OR Netherlands Antilles[tw] OR New Caledonia[tw] OR Nicaragua[tw] OR Niger[tw] OR Nigeria[tw] OR Northern Mariana Islands[tw] OR Oman[tw] OR Muscat[tw] OR Pakistan[tw] OR Palau[tw] OR Palestine[tw] OR Panama[tw] OR Paraguay[tw] OR Peru[tw] OR Philippines[tw] OR Philipines[tw] OR Phillipines[tw] OR Phillippines[tw] OR Poland[tw] OR Portugal[tw] OR Puerto Rico[tw] OR Romania[tw] OR Rumania[tw] OR Roumania[tw] OR Russia[tw] OR Russian[tw] OR Rwanda[tw] OR Ruanda[tw] OR Saint Kitts[tw] OR St Kitts[tw] OR Nevis[tw] OR Saint Lucia[tw] OR St Lucia[tw] OR Saint Vincent[tw] OR St Vincent[tw] OR Grenadines[tw] OR Samoa[tw] OR Samoan Islands[tw] OR (Navigator[All Fields] AND Island[tw]) OR (Navigator[All Fields] AND Islands[tw]) OR Sao Tome[tw] OR Saudi Arabia[tw] OR Senegal[tw] OR Serbia[tw] OR Montenegro[tw] OR Seychelles[tw] OR Sierra Leone[tw] OR Slovenia[tw] OR Sri Lanka[tw] OR Ceylon[tw] OR Solomon Islands[tw] OR Somalia[tw] OR Sudan[tw] OR Suriname[tw] OR Surinam[tw] OR Swaziland[tw] OR Syria[tw] OR Tajikistan[tw] OR Tadzhikistan[tw] OR Tadjikistan[tw] OR Tadzhik[tw] OR Tanzania[tw] OR Thailand[tw] OR Togo[tw] OR Togolese Republic[tw] OR Tonga[tw] OR Trinidad[tw] OR Tobago[tw] OR Tunisia[tw] OR Turkey[tw] OR Turkmenistan[tw] OR Turkmen[tw] OR Uganda[tw] OR Ukraine[tw] OR Uruguay[tw] OR USSR[tw] OR Soviet Union[tw] OR Union of Soviet Socialist Republics[tw] OR Uzbekistan[tw] OR Uzbek[All Fields] OR Vanuatu[tw] OR New Hebrides[tw] OR Venezuela[tw] OR Vietnam[tw] OR Viet Nam[tw] OR West Bank[tw] OR Yemen[tw] OR Yugoslavia[tw] OR Zambia[tw] OR Zimbabwe[tw] OR Rhodesia[tw])) OR (Africa[tw] OR Asia[tw] OR Caribbean[tw] OR West Indies[tw] OR South America[tw] OR Latin America[tw] OR Central America[tw] OR Afghanistan[tw] OR Albania[tw] OR Algeria[tw] OR Angola[tw] OR Antigua[tw] OR Barbuda[tw] OR Argentina[tw] OR Armenia[tw] OR Armenian[tw] OR Aruba[tw] OR Azerbaijan[tw] OR Bahrain[tw] OR Bangladesh[tw] OR Barbados[tw] OR Benin[tw] OR Byelarus[tw] OR Byelorussian[tw] OR Belarus[tw] OR Belorussian[tw] OR Belorussia[tw] OR Belize[tw] OR Bhutan[tw] OR Bolivia[tw] OR Bosnia[tw] OR Herzegovina[tw] OR Hercegovina[tw] OR Botswana[tw] OR Brasil[tw] OR Brazil[tw] OR Bulgaria[tw] OR Burkina Faso[tw] OR Burkina Fasso[tw] OR Upper Volta[tw] OR Burundi[tw] OR Urundi[tw] OR Cambodia[tw] OR Khmer Republic[tw] OR Kampuchea[tw] OR Cameroon[tw] OR Cameroons[tw] OR Cameron[tw] OR Cape Verde[tw] OR Central African Republic[tw] OR Chad[tw] OR Chile[tw] OR China[tw] OR Colombia[tw] OR Comoros[tw] OR Comoro Islands[tw] OR Comores[tw] OR Mayotte[tw] OR Congo[tw] OR Zaire[tw] OR Costa Rica[tw] OR Cote d'Ivoire[tw] OR Ivory Coast[tw] OR Croatia[tw] OR Cuba[tw] OR Cyprus[tw] OR Czechoslovakia[tw] OR Czech Republic[tw] OR Slovakia[tw] OR Slovak Republic[tw] OR Djibouti[tw] OR French Somaliland[tw] OR Dominica[tw] OR Dominican Republic[tw] OR East Timor[tw] OR (East[All Fields] AND Timur[tw]) OR Timor Leste[tw] OR Ecuador[tw] OR Egypt[tw] OR United Arab Republic[tw] OR El Salvador[tw] OR Eritrea[tw] OR Estonia[tw] OR Ethiopia[tw] OR Fiji[tw] OR Gabon[tw] OR Gabonese Republic[tw] OR Gambia[tw] OR Gaza[tw] OR Georgia Republic[tw] OR Georgian Republic[tw] OR Ghana[tw] OR Gold Coast[tw] OR Greece[tw] OR Grenada[tw] OR Guatemala[tw] OR Guinea[tw] OR Guam[tw] OR Guiana[tw] OR Guyana[tw] OR Haiti[tw] OR Honduras[tw] OR Hungary[tw] OR India[tw] OR Maldives[tw] OR Indonesia[tw] OR Iran[tw] OR Iraq[tw] OR Isle of Man[tw] OR Jamaica[tw] OR Jordan[tw] OR Kazakhstan[tw] OR Kazakh[tw] OR Kenya[tw] OR Kiribati[tw] OR Korea[tw] OR Kosovo[tw] OR Kyrgyzstan[tw] OR Kirghizia[tw] OR Kyrgyz Republic[tw] OR Kirghiz[tw] OR Kirgizstan[tw] OR "Lao PDR"[tw] OR Laos[tw] OR Latvia[tw] OR Lebanon[tw] OR Lesotho[tw] OR Basutoland[tw] OR Liberia[tw] OR Libya[tw] OR Lithuania[tw])) OR ("developing country"[tw] OR "developing countries"[tw] OR "developing nation"[tw] OR "developing nations"[tw] OR "developing population"[tw] OR "developing populations"[tw] OR "developing world"[tw] OR "less developed country"[tw] OR "less developed countries"[tw] OR "less developed nation"[tw] OR "less developed nations"[tw] OR "less developed world"[tw] OR "lesser developed countries"[tw] OR "lesser developed nations"[tw] OR "under developed country"[tw] OR "under developed countries"[tw] OR "under developed nations"[tw] OR "under developed world"[tw] OR "underdeveloped country"[tw] OR "underdeveloped countries"[tw] OR "underdeveloped nations"[tw] OR "underdeveloped population"[tw] OR "underdeveloped world"[tw] OR "middle income country"[tw] OR "middle income countries"[tw] OR "middle income nation"[tw] OR "middle income nations"[tw] OR "middle income population"[tw] OR "middle income populations"[tw] OR "low income country"[tw] OR "low income countries"[tw] OR "low income nations"[tw] OR "low income population"[tw] OR "low income populations"[tw] OR "lower income country"[tw] OR "lower income countries"[tw] OR "lower income nations"[tw] OR "lower income population"[tw] OR "lower income populations"[tw] OR "underserved countries"[tw] OR "underserved nations"[tw] OR "underserved population"[tw] OR "underserved populations"[tw] OR "under served population"[tw] OR "under served populations"[tw] OR "deprived countries"[tw] OR "deprived population"[tw] OR "deprived populations"[tw] OR "poor country"[tw] OR "poor countries"[tw] OR "poor nation"[tw] OR "poor nations"[tw] OR "poor population"[tw] OR "poor populations"[tw] OR "poor world"[tw] OR "poorer countries"[tw] OR "poorer nations"[tw] OR "poorer population"[tw] OR "poorer populations"[tw] OR "developing economy"[tw] OR "developing economies"[tw] OR "less developed economies"[tw] OR "underdeveloped economies"[tw] OR "middle income economies"[tw] OR "low income economy"[tw] OR "low income economies"[tw] OR "low gdp"[tw] OR "low gnp"[tw] OR "low gross domestic"[tw] OR "low gross national"[tw] OR "lower gdp"[tw] OR "lower gross domestic"[tw] OR lmic[tw] OR lmics[tw] OR "third world"[tw] OR "lami country"[tw] OR "lami countries"[tw] OR "transitional country"[tw] OR "transitional countries"[tw]))

| **PubMed search syntax**  [tiab] denotes a word in the title or abstract;  [mh] denotes a Medical Subject Heading (MeSH) term (‘exploded’);  [mesh: noexp] denotes a Medical Subject Heading (MeSH) term (not ‘exploded’);  [ti] denotes a word in the title.  [ot] other term  [pl] place of publication  Source: (Higgins, Green 2008) |
| --- |

**EMERALD (7545)**

(Organi?ational Culture OR institutional culture AND health sector reform* OR Health Care Reform* OR Health Polic* OR "health system strengthening interventions" OR universal health coverage OR "user fee removal" OR "user fees" OR "pay for performance" OR "performance based financing" OR health sector strateg* OR "health sector reform" OR "health reform" OR decentralization OR decentralisation OR politics OR contracting out OR outsourc* OR public private partnerships OR comprehensive primary health care AND implement* AND Developing Countr* OR Africa OR Asia OR Latin America OR Caribbean OR Pacific OR Middle East OR East Europe OR transitional countr* OR low income countr* OR middle income countr* OR LMIC OR LMICs)

FILTERS APPLIED:

Type Research paper

Publication Date: 01/01/2000 - 12/31/2015

| [*] indicates truncation |
| --- |

**Africa-Wide Information (9)**

(Organi?ational Culture OR institutional culture AND health sector reform* OR Health Care Reform* OR Health Polic* OR "health system strengthening interventions" OR universal health coverage OR "user fee removal" OR "user fees" OR "pay for performance" OR "performance based financing" OR health sector strateg* OR "health sector reform" OR "health reform" OR decentralization OR decentralisation OR politics OR contracting out OR outsourc* OR public private partnerships OR comprehensive primary health care AND implement* AND Developing Countr* OR Africa OR Asia OR Latin America OR Caribbean OR Pacific OR Middle East OR East Europe OR transitional countr* OR low income countr* OR middle income countr* OR LMIC OR LMICs)

SEARCH OPTIONS

Search modes - Boolean/Phrase

Limit your results – Year published: 20000101-20151231

**CINAHL (3)**

(Organi?ational Culture OR institutional culture AND health sector reform* OR Health Care Reform* OR Health Polic* OR "health system strengthening interventions" OR universal health coverage OR "user fee removal" OR "user fees" OR "pay for performance" OR "performance based financing" OR health sector strateg* OR "health sector reform" OR "health reform" OR decentralization OR decentralisation OR politics OR contracting out OR outsourc* OR public private partnerships OR comprehensive primary health care AND implement* AND Developing Countr* OR Africa OR Asia OR Latin America OR Caribbean OR Pacific OR Middle East OR East Europe OR transitional countr* OR low income countr* OR middle income countr* OR LMIC OR LMICs)

SEARCH OPTIONS

Search modes - Boolean/Phrase

Limit your results – Year published: 20000101-20151231

**ECONLIT (4)**

(Organi?ational Culture OR institutional culture AND health sector reform* OR Health Care Reform* OR Health Polic* OR "health system strengthening interventions" OR universal health coverage OR "user fee removal" OR "user fees" OR "pay for performance" OR "performance based financing" OR health sector strateg* OR "health sector reform" OR "health reform" OR decentralization OR decentralisation OR politics OR contracting out OR outsourc* OR public private partnerships OR comprehensive primary health care AND implement* AND Developing Countr* OR Africa OR Asia OR Latin America OR Caribbean OR Pacific OR Middle East OR East Europe OR transitional countr* OR low income countr* OR middle income countr* OR LMIC OR LMICs)

SEARCH OPTIONS

Search modes - Boolean/Phrase

Limit your results – Year published: 20000101-20151231

**PsycINFO (8)**

(Organi?ational Culture OR institutional culture AND health sector reform* OR Health Care Reform* OR Health Polic* OR "health system strengthening interventions" OR universal health coverage OR "user fee removal" OR "user fees" OR "pay for performance" OR "performance based financing" OR health sector strateg* OR "health sector reform" OR "health reform" OR decentralization OR decentralisation OR politics OR contracting out OR outsourc* OR public private partnerships OR comprehensive primary health care AND implement* AND Developing Countr* OR Africa OR Asia OR Latin America OR Caribbean OR Pacific OR Middle East OR East Europe OR transitional countr* OR low income countr* OR middle income countr* OR LMIC OR LMICs)

SEARCH OPTIONS

Search modes - Boolean/Phrase

Limit your results – Year published: 20000101-20151231

**SocINDEX WITH FULL TEXT (2)**

(Organi?ational Culture OR institutional culture AND health sector reform* OR Health Care Reform* OR Health Polic* OR "health system strengthening interventions" OR universal health coverage OR "user fee removal" OR "user fees" OR "pay for performance" OR "performance based financing" OR health sector strateg* OR "health sector reform" OR "health reform" OR decentralization OR decentralisation OR politics OR contracting out OR outsourc* OR public private partnerships OR comprehensive primary health care AND implement* AND Developing Countr* OR Africa OR Asia OR Latin America OR Caribbean OR Pacific OR Middle East OR East Europe OR transitional countr* OR low income countr* OR middle income countr* OR LMIC OR LMICs)

SEARCH OPTIONS

Search modes - Boolean/Phrase

Limit your results – Year published: 20000101-20151231

**SCOPUS SEARCH STRATEGY (55)**

((TITLE-ABS-KEY(Decentralization) OR TITLE-ABS-KEY(Decentralisation) OR TITLE-ABS-KEY(user fee removal ) OR TITLE-ABS-KEY(user fees ) OR TITLE-ABS-KEY(health sector reforms ) OR TITLE-ABS-KEY(Health Care Reform ) OR TITLE-ABS-KEY("Health Policy") OR TITLE-ABS-KEY(pay for performance )OR TITLE-ABS-KEY(performance based financing ) OR TITLE-ABS-KEY(health sector strateg* ) OR TITLE-ABS-KEY(health system reform ) OR TITLE-ABS-KEY(health reform ) OR TITLE-ABS-KEY(universal health coverage ) OR TITLE-ABS-KEY(comprehensive primary health care) OR TITLE-ABS-KEY(outsourc*) OR TITLE-ABS-KEY(public private partnerships) OR TITLE-ABS-KEY(health system strengthening interventions ))) AND TITLE-ABS-KEY("Organizational Culture" ) OR TITLE-ABS-KEY("Organisational Culture" ) OR TITLE-ABS-KEY("Institutional Culture" ) AND TITLE-ABS-KEY(implement* ) AND (((TITLE-ABS-KEY(underserved OR under served ) OR TITLE-ABS-KEY(deprived OR poor*) OR TITLE-ABS-KEY(low AND middle AND countr* ) OR TITLE-ABS-KEY(lmic OR lmics ) OR TITLE-ABS-KEY(third world OR lami countr*) OR TITLE-ABS-KEY(transitional countr*))) OR (((TITLE-ABS-KEY(Developing Country ) OR TITLE-ABS-KEY(Africa) OR TITLE-ABS-KEY(Asia ) OR TITLE-ABS-KEY( Caribbean ) OR TITLE-ABS-KEY(West Indies ) OR TITLE-ABS-KEY(South America ) OR TITLE-ABS-KEY(Latin America) OR TITLE-ABS-KEY(Central America) OR TITLE-ABS-KEY(Afghanistan) OR TITLE-ABS-KEY(Albania) OR TITLE-ABS-KEY(Algeria) OR TITLE-ABS-KEY(Angola) OR TITLE-ABS-KEY(Antigua) OR TITLE-ABS-KEY(Barbuda) OR TITLE-ABS-KEY(Benin) OR TITLE-ABS-KEY(Byelarus) OR TITLE-ABS-KEY(Byelorussian ) OR TITLE-ABS-KEY( Belarus ) OR TITLE-ABS-KEY(Belorussian) OR TITLE-ABS-KEY(Belorussia))) OR ((TITLE-ABS-KEY(Belize ) OR TITLE-ABS-KEY(Bhutan) OR TITLE-ABS-KEY(Bolivia ) OR TITLE-ABS-KEY(Bosnia) OR TITLE-ABS-KEY(Herzegovina) OR TITLE-ABS-KEY(Hercegovina) OR TITLE-ABS-KEY(Botswana) OR TITLE-ABS-KEY(Brasil ) OR TITLE-ABS-KEY(Brazil) OR TITLE-ABS-KEY(Bulgaria ) OR TITLE-ABS-KEY(Burkina Faso ) OR TITLE-ABS-KEY(Burkina Fasso ) OR TITLE-ABS-KEY(Upper Volta ) OR TITLE-ABS-KEY(Burundi) OR TITLE-ABS-KEY(Urundi) OR TITLE-ABS-KEY(Cambodia ) OR TITLE-ABS-KEY(Khmer Republic) OR TITLE-ABS-KEY(Kampuchea) OR TITLE-ABS-KEY(Cameroon) OR TITLE-ABS-KEY(Cameroons))) OR ((TITLE-ABS-KEY(Cameron) OR TITLE-ABS-KEY(Camerons ) OR TITLE-ABS-KEY(Cape Verde ) OR TITLE-ABS-KEY(Central African Republic) OR TITLE-ABS-KEY(Chad) OR TITLE-ABS-KEY(Chile) OR TITLE-ABS-KEY(China) OR TITLE-ABS-KEY(Colombia) OR TITLE-ABS-KEY(Comoros) OR TITLE-ABS-KEY(Comoro Islands ) OR TITLE-ABS-KEY(Comores) OR TITLE-ABS-KEY(Mayotte) OR TITLE-ABS-KEY(Congo) OR TITLE-ABS-KEY(Zaire) OR TITLE-ABS-KEY(Costa Rica ) OR TITLE-ABS-KEY(Cote d'Ivoire ) OR TITLE-ABS-KEY(Ivory Coast ) OR TITLE-ABS-KEY(Croatia) OR TITLE-ABS-KEY(Cuba ) OR TITLE-ABS-KEY(Cyprus))) OR ((TITLE-ABS-KEY(Czechoslovakia ) OR TITLE-ABS-KEY(Czech Republic ) OR TITLE-ABS-KEY(Slovakia) OR TITLE-ABS-KEY(Slovak Republic ) OR TITLE-ABS-KEY(Djibouti) OR TITLE-ABS-KEY(French Somaliland) OR TITLE-ABS-KEY(Dominica) OR TITLE-ABS-KEY(Dominican Republic OR East Timor) OR TITLE-ABS-KEY(East Timur OR Timor Leste ) OR TITLE-ABS-KEY(Ecuador OR Egypt ) OR TITLE-ABS-KEY(United Arab Republic OR El Salvador) OR TITLE-ABS-KEY(Eritrea OR Estonia ) OR TITLE-ABS-KEY(Ethiopia OR Fiji ) OR TITLE-ABS-KEY(Gabon OR Gabonese Republic ) OR TITLE-ABS-KEY(Gambia OR Gaza) OR TITLE-ABS-KEY(Georgia Republic OR Georgian Republic ) OR TITLE-ABS-KEY(Ghana OR Gold Coast ) OR TITLE-ABS-KEY(Greece OR Grenada ) OR TITLE-ABS-KEY(Guatemala OR Guinea ) OR TITLE-ABS-KEY(Guam OR Guiana ))) OR ((TITLE-ABS-KEY(Guyana OR Haiti ) OR TITLE-ABS-KEY(Honduras OR Hungary ) OR TITLE-ABS-KEY(India OR Maldives ) OR TITLE-ABS-KEY(Indonesia OR Iran ) OR TITLE-ABS-KEY(Iraq OR Isle of Man ) OR TITLE-ABS-KEY(Jamaica OR Jordan ) OR TITLE-ABS-KEY(Kazakhstan OR Kazakh ) OR TITLE-ABS-KEY(Kenya OR Kiribati) OR TITLE-ABS-KEY(Korea OR Kosovo ) OR TITLE-ABS-KEY(Kyrgyzstan OR Kirghizia ) OR TITLE-ABS-KEY(Kyrgyz Republic OR Kirghiz ) OR TITLE-ABS-KEY(Kirgizstan OR Lao PDR ) OR TITLE-ABS-KEY(Laos OR Latvia ) OR TITLE-ABS-KEY(Lebanon OR Lesotho ) OR TITLE-ABS-KEY(Basutoland OR Liberia ) OR TITLE-ABS-KEY(Libya OR Lithuania ) OR TITLE-ABS-KEY(Macedonia OR Madagascar ) OR TITLE-ABS-KEY(Malagasy Republic OR Malaysia ) OR TITLE-ABS-KEY(Malaya OR Malay ) OR TITLE-ABS-KEY(Sabah OR Sarawak ))) OR ((TITLE-ABS-KEY(Malawi OR Nyasaland ) OR TITLE-ABS-KEY(Mali OR Malta ) OR TITLE-ABS-KEY(Marshall Islands OR Mauritania ) OR TITLE-ABS-KEY(Mauritius OR Agalega Islands ) OR TITLE-ABS-KEY(Mexico OR Micronesia ) OR TITLE-ABS-KEY(Middle East OR Moldova ) OR TITLE-ABS-KEY(Moldovia OR Moldovian ) OR TITLE-ABS-KEY(Mongolia OR Montenegro ) OR TITLE-ABS-KEY(Morocco OR Ifni ) OR TITLE-ABS-KEY(Mozambique OR Myanmar ) OR TITLE-ABS-KEY(Myanma OR Burma ) OR TITLE-ABS-KEY(Namibia OR Nepal ) OR TITLE-ABS-KEY(Netherlands Antilles OR New Caledonia ) OR TITLE-ABS-KEY(Nicaragua OR Niger ) OR TITLE-ABS-KEY(Nigeria OR Northern Mariana Islands ) OR TITLE-ABS-KEY(Oman OR Muscat ) OR TITLE-ABS-KEY(Pakistan OR Palau ) OR TITLE-ABS-KEY(Palestine OR Panama ) OR TITLE-ABS-KEY(Paraguay OR Peru ) OR TITLE-ABS-KEY(Philippines OR Philipines ))) OR ((TITLE-ABS-KEY(Phillipines OR Phillippines ) OR TITLE-ABS-KEY(Poland OR Portugal ) OR TITLE-ABS-KEY(Puerto Rico OR Romania ) OR TITLE-ABS-KEY(Rumania OR Roumania ) OR TITLE-ABS-KEY(Russia OR Russian) OR TITLE-ABS-KEY(Rwanda OR Ruanda ) OR TITLE-ABS-KEY(Saint Kitts OR St Kitts) OR TITLE-ABS-KEY(Nevis OR Saint Lucia ) OR TITLE-ABS-KEY(St Lucia OR Saint Vincent ) OR TITLE-ABS-KEY(St Vincent OR Grenadines) OR TITLE-ABS-KEY(Samoa OR Samoan Islands ) OR TITLE-ABS-KEY(Navigator Island OR Navigator Islands ) OR TITLE-ABS-KEY(Sao Tome OR Saudi Arabia ) OR TITLE-ABS-KEY(Senegal OR Serbia ) OR TITLE-ABS-KEY(Montenegro OR Seychelles ) OR TITLE-ABS-KEY(Sierra Leone OR Slovenia ) OR TITLE-ABS-KEY(Sri Lanka OR Ceylon) OR TITLE-ABS-KEY(Solomon Islands OR Somalia ) OR TITLE-ABS-KEY(South Africa OR Sudan ) OR TITLE-ABS-KEY(Suriname OR Surinam ))) OR ((TITLE-ABS-KEY(Swaziland OR Syria ) OR TITLE-ABS-KEY(Tajikistan OR Tadzhikistan ) OR TITLE-ABS-KEY(Tadjikistan OR Tadzhik ) OR TITLE-ABS-KEY(Tanzania OR Thailand) OR TITLE-ABS-KEY(Togo OR Togolese Republic ) OR TITLE-ABS-KEY(Tonga OR Trinidad ) OR TITLE-ABS-KEY(Tobago OR Tunisia ) OR TITLE-ABS-KEY(Turkey OR Turkmenistan ) OR TITLE-ABS-KEY(Turkmen OR Uganda ) OR TITLE-ABS-KEY(Ukraine OR Uruguay ) OR TITLE-ABS-KEY(USSR OR Soviet Union ) OR TITLE-ABS-KEY(Union of Soviet Socialist Republics OR Uzbekistan ) OR TITLE-ABS-KEY(Uzbek OR Vanuatu ) OR TITLE-ABS-KEY(New Hebrides OR Venezuela ) OR TITLE-ABS-KEY(Vietnam OR Viet Nam ) OR TITLE-ABS-KEY(West Bank OR Yemen ) OR TITLE-ABS-KEY(Yugoslavia OR Zambia ) OR TITLE-ABS-KEY(Zimbabwe OR Rhodesia) OR TITLE-ABS-KEY(developing OR less* developed ) OR TITLE-ABS-KEY(under developed OR underdeveloped ) OR TITLE-ABS-KEY(middle income OR low* income ))))) AND ( LIMIT-TO(PUBYEAR,2015) OR LIMIT-TO(PUBYEAR,2014) OR LIMIT-TO(PUBYEAR,2013) OR LIMIT-TO(PUBYEAR,2012) OR LIMIT-TO(PUBYEAR,2011) OR LIMIT-TO(PUBYEAR,2010) OR LIMIT-TO(PUBYEAR,2009) OR LIMIT-TO(PUBYEAR,2008) OR LIMIT-TO(PUBYEAR,2007) OR LIMIT-TO(PUBYEAR,2006) OR LIMIT-TO(PUBYEAR,2005) OR LIMIT-TO(PUBYEAR,2004) OR LIMIT-TO(PUBYEAR,2003) OR LIMIT-TO(PUBYEAR,2002) OR LIMIT-TO(PUBYEAR,2001) OR LIMIT-TO(PUBYEAR,2000) ) AND ( LIMIT-TO(LANGUAGE,"English" ) )

Table 1: Database and date of last search

| **Name of database** | **Date of last search** |
| --- | --- |
| Africa-Wide information | 29.12.2015 |
| Cumulative Index of Nursing and Allied Health Literature (CINAHL) | 28.12.2015 |
| Econlit | 28.12.2015 |
| Emerald | 29.12.2015 |
| PsycINFO | 28.12.2015 |
| PubMed | 28.12.2015 |
| Scopus | 31.12.2015 |
| SocINDEX with full text | 28.12.2015 |
