# Supplementary material for: Influence of organisational culture on the implementation of health sector reforms in low- and middle-income countries: a qualitative interpretive review
Source: Glob Health Action. 2018 May 11;11(1):1462579. doi: 10.1080/16549716.2018.1462579 (PMC5954479; doi:10.1080/16549716.2018.1462579)
Supplement: Supplementary material [file ZGHA_A_1462579_SM0646.zip › Supplementary material 2-Articles included in the review.docx]

# **S2. Summary of the articles included in the review**

| **Title of article, Name of author, publication year** | **Geographical region** | **Type of health sector reform** | **Methods**  **Study setting** | **Brief overview of the article** |
| --- | --- | --- | --- | --- |
| **Analysis of the perception of institutional culture for health sector reform in Nigeria[38]** | Nigeria | Comprehensive health sector reforms that aim to strengthen preventive and curative primary health care services and enhance provision of quality and affordable health services | Cross-sectional study using close-ended questionnaires  4 hospitals: Two public sector university hospitals, one public sector hospital and one not-for- profit mission hospital | Following the introduction of a comprehensive health sector reform programme by Nigeria’s Ministry of Health in 2005, this study explores the perceptions of organisational culture held by all the professional and managerial members in four hospitals and how the culture influences the implementation of this reform. The two dimensions of culture that were explored in this study are leadership and character whereby character refers to the identity of the organisation that distinguishes it from other organisations. |
| **Barriers to implementing health sector administrative decentralisation in Ghana**  **A study of the Nkwanta district health management team [36]** | Ghana | Decentralisation | Qualitative study conducted using semi-structured individual and group interviews with managers and junior workers from Nkwanta district health management team who had been involved in the implementation process | This empirical study seeks to understand how health workers’ level of knowledge and understanding of the reform objectives influences the implementation of the reforms. It also seeks to identify organisational factors that influence the implementation of the decentralisation policy within the local district health system |
| **Communication challenges in implementing health sector decentralisation at district level in Ghana. A study of health workforce and stakeholder opinions from three district health administrations [35]** | Ghana | Decentralisation | Qualitative study using in-depth interviews with district public health and local government officials, private health care providers and health-related non-government organisations in three districts: Dangme West, Sekyere West and Tamale districts | This study examines how internal communication of the decentralisation policy and its objectives influences the implementation of the decentralisation policy at the district level. It focuses on identifying challenges in communication by exploring managers’, health workers’ and stakeholders’ perceptions of the channels, adequacy and effect of shared information. |
| **Global rights and state activism: Reflections on civil society—State partnerships in health in NW India [41]** | India | Civil Society Organisations (CSOs) and State Partnerships in development programmes under the National Rural Health Mission | Multi-site Ethnographic study over a one year period (July 2009- June 2010).  Data collection was done through questionnaires, interviews (informal and semi-structured), focus group discussions and participant observation in meetings and trainings with members of the CSOs working in Rajasthan state, India | This study seeks to understand how the CSOs-State partnerships function to promote rural health by drawing on the perspectives of the CSOs members. It also seeks to understand how the differences in culture and values between the two organisations affect the sustainability of these partnerships |
| **Going down to the local: incorporating social organisation and political culture into assessments of decentralized health care [42]** | Brazil | Decentralisation | Ethnographic case studies in three district health systems (metropolitan, rural and urban) in Ceara State.    Data was collected through formal open interviews, observations, informal conversations, field notes and document review | This study seeks to understand how the dimensions of the social organisation and political culture-which form aspects of the local environment- influence the implementation of the decentralisation policy and achievement of the reform objectives across three district health systems. |
| **Implementing decentralised**  **management in Ghana**  **The experience of the Sekyere West District**  **health administration [37]** | Ghana | Decentralisation of health services and programmes | A case study approach using in-depth interviews and focus group discussions. Participants included district public health officials, local government officials, private health care providers and non-government organisations who had worked in Sekyere West District for at least two years. | This study explores the barriers to the implementation of the decentralisation policy in Ghana by drawing on the perceptions of health managers, health workers and stakeholders. |
| **Political cultures, health systems and health policy [43]** | Brazil | Decentralisation | Ethnographic case studies carried out within three districts (rural, urban and metropolitan). | This study explores the influence of political culture and other aspects of the social environment on the implementation and performance of the decentralisation policy across three district health systems in Brazil. |
| **Restructuring a ministry of health – an issue of structure and process: a case study from Uganda [39]** | Uganda | Health system restructuring in the context of Decentralisation | Qualitative study involving: participant observation, interviews with civil servants within the Ministry of Health and Donors, review of documents and, discussions with district officials | This study assesses whether the restructuring of the Ministry of health supported the decentralisation policy. It also analyses the factors that challenged the implementation of the National Health Policies in the context of decentralisation |
